# A zinc-free alternative ribosomal protein RpsN.2 confers survival advantage to group A streptococcus during Zn scarcity

**DOI:** 10.1128/iai.00442-25

**Published:** 2025-11-18

**Authors:** Subhasree Saha, Aswin Thacharodi, Dieu Linh Nguyen, Nishanth Makthal, Charles Bernard, Muthiah Kumaraswami

**Affiliations:** 1 Center for Molecular and Translational Human Infectious Diseases Research, Houston Methodist Research Institute 167626 , Houston, Texas, USA; 2 Department of Pathology and Genomic Medicine, Houston Methodist Hospital 23534 , Houston, Texas, USA; 3 Institut Pasteur, Université de Paris, Centre nationale de la recherche scientifique (CNRS), Unité mixte de recherche (UMR) 3525, Microbial Evolutionary Genomics 27058, Paris, France; University of Illinois Chicago, Chicago, Illinois, USA

**Keywords:** Zn scarcity, oral pathogen, commensal, nutritional immunity, rpsN.2

## Abstract

Infection induces unfavorable environments in the host that can be detrimental to the survival of commensal and pathogenic bacteria. Although the adaptive strategies employed by pathogenic bacteria to overcome harsh environments are characterized, similar capabilities of the commensal bacteria to survive in hostile host niches during infection remain understudied. The human oral pathogen group A streptococcus (GAS) encounters host-induced zinc (Zn) limitation at infection sites that limits bacterial proliferation. However, GAS employs the Zn-sensing transcription regulator AdcR to monitor Zn levels and evades host-imposed Zn scarcity by upregulating the AdcR regulon. To elucidate the adaptive responses of oropharyngeal commensal streptococci to Zn scarcity, we analyzed the oropharyngeal pathogenic and commensal streptococcal genomes for the presence of the AdcR regulon. GAS has the full repertoire of the AdcR regulon that includes *adcR*, Zn importer *adcABC*, extracellular Zn binding proteins *adcAII* and *Pht*, and Zn-free alternative ribosomal S14 subunit, *rpsN.2*. Contrarily, except for the conserved presence of *adcR* and *adcABC*, the oropharyngeal commensal streptococci varied in the composition of the AdcR regulon. Specifically, the gene encoding *rpsN.2* was absent in the screened commensal streptococcal genomes. We further demonstrated that *rpsN.2* is critical for the survival of GAS in Zn-limiting environments including human saliva, whereas the commensal *Streptococcus vestibularis* that lacks several components of the AdcR regulon, including *rpsN.2*, is defective in survival in Zn-deficient conditions. Together, we identified a pathogen-specific adaptive strategy that aids evasion of host-imposed Zn limitation and confers survival advantage over oropharyngeal commensal streptococci during Zn scarcity.

## INTRODUCTION

Pathogenic bacteria survive among host-associated beneficial microbial communities in non-sterile host niches. Infection by pathogens and infection-related host inflammation responses can be detrimental to beneficial microbes and cause perturbations in the composition of microbial communities (1–4). However, pathogenic bacteria can adapt to inflammation-associated environmental changes and survive successfully in inflamed tissues (5–7). The elucidation of adaptive strategies that aid the bacterial evasion of host innate immune responses and support the survival of pathogens in inflamed tissues may help identify new antimicrobial targets.

A major inflammation-dependent environmental change involves the alterations in the availability of transition metals, such as iron (Fe), manganese (Mn), and zinc (Zn), at infection sites. Neutrophils are deployed to infection sites as a component of host innate immune responses and exert antimicrobial activity via several mechanisms. The neutrophils release calprotectin (CP), a heterodimer of S100A8 and S100A9 proteins, at the infection sites, and the extracellular CP scavenges Fe, Mn, and Zn from the host-pathogen interfaces (8–16). Given that Zn, Mn, and Fe are essential micronutrients required for bacterial survival and proliferation (17–19), metal limitation by CP is a critical component of host defenses against pathogenic bacteria (8–16). However, pathogens evolved evasive mechanisms to overcome host-imposed metal scarcity and colonize successfully (10, 11, 14, 15). The well-characterized bacterial adaptive response to host-imposed metal deficiency involves the upregulation of genes encoding high-affinity metal uptake systems and the procurement of extracellular metals to sustain bacterial growth (10, 11, 14, 15, 20). Contrary to this, alterations in metal levels have a negative impact on the composition of beneficial microbial communities, and such shifts in host-associated microbial community structure can impact host susceptibility to infections (21–23).

Group A streptococcus (GAS), also known as *Streptococcus pyogenes*, is an exclusive human oral pathogen that causes major disease manifestations, including pharyngitis and invasive infections (24–27). GAS encounters host-derived CP during infection and remains susceptible to CP-mediated Zn and Mn limitation (15). To survive in Zn-limited host environments, GAS deploys the Zn-sensing transcription regulator AdcR, which senses Zn scarcity and orchestrates the upregulation of the AdcR regulon. The AdcR regulon includes genes encoding the Zn importer *adcABC*, extracellular Zn-binding proteins *adcAII* an polyhistidinee triad proteins *phtD* and *phtY*, and the alternative Zn-free ribosomal S14 subunit *rpsN.2* (15, 28–30). The inactivation of each of the aforementioned genes reduced GAS survival and virulence in the presence of CP but was dispensable in the absence of CP, indicating their roles in evading CP-mediated Zn limitation during infection (15, 28, 30–34). Similarly, Zn sensing by AdcR, Zn-dependent regulation of the AdcR regulon, and their contribution to bacterial virulence have been demonstrated in other streptococcal pathogens (35–41). Contrary to these observations in pathogenic streptococci, the adaptive responses and potential impact of Zn scarcity or CP-mediated Zn limitation on the survival of oropharyngeal commensal streptococci remain largely uncharacterized.

To compare the adaptive strategies of oropharyngeal commensal and pathogenic streptococci to Zn scarcity, we performed bioinformatic analyses of the genomes of oropharyngeal streptococci to determine the presence of the components of the AdcR regulon. We show that, except for the conserved presence of paralogs of the Zn importer *adcABC* in both commensal and pathogenic oropharyngeal streptococci, the oropharyngeal commensal streptococcal genomes vary in the presence of *adcAII*, *pht*, and *rpsN.2*. Intriguingly, the gene encoding *rpsN.2* was absent in the genomes of all screened oropharyngeal commensal streptococci, suggesting that *rpsN.2* is a critical component of pathogenic streptococcal adaptive responses to evade host-imposed Zn scarcity. In accordance with these findings, we further demonstrated that *rpsN.2* plays a key role in GAS survival in Zn scant human saliva and CP resistance. Our characterization of *S. vestibularis*, a representative oropharyngeal commensal streptococcus, showed that *S. vestibularis* is more sensitive to Zn scarcity, defective in survival in human saliva, and susceptible to CP-mediated Zn limitation compared with that of GAS. Based on these observations, we propose that *rpsN.2* confers a survival advantage to oropharyngeal pathogenic streptococci in Zn-limited niches in the human oral cavity and contributes significantly to disease pathogenesis.

## RESULTS

### Oropharyngeal commensal streptococci lack the gene encoding *rpsN.2*

To compare the survival strategies used by the oropharyngeal commensal and pathogenic streptococci during Zn scarcity, we screened the oropharyngeal streptococcal genomes for the presence of components of the AdcR regulon. Since the members of the genus *Streptococcus* are predominant in the healthy human oral microbiome and are closely related to GAS (42–48), it is likely that major interactions between commensal streptococci and GAS occur in the oral cavity. Among the eight well-represented clades of the genus *Streptococcus*, species from five clades (Sanguinis, Mitis, Anginosus, Salivarius, and Mutans) are prominent within healthy oral sites (47, 48). Thus, we chose the reference genomes from the members of each clade, *S. sanguinis*, *S. gordonii*, and *S. cristatus* from Sanguinis, *S. infantis, S. oralis, S. pseudopneumoniae, S. parasanguinis*, and *S. mitis* from Mitis, *S. anginosus* and *S. intermedius* from Anginosus, *S. salivarius* and *S. vestibularis* from Salivarius, and *S. mutans* from Mutans, for the analyses of the AdcR regulon genes (Table S1). We have also included *S. pneumoniae*, a major nasopharyngeal human pathogen, in the analyses. Given that the GAS AdcR regulon genes are well characterized for their roles in bacterial survival during Zn scarcity, CP resistance, and bacterial virulence (15, 28–30), we analyzed the annotated proteomes of oropharyngeal commensal streptococci using the experimentally verified protein sequences corresponding to the gene products of the GAS AdcR regulon (Tables S2 to S4).

**Fig 1:**
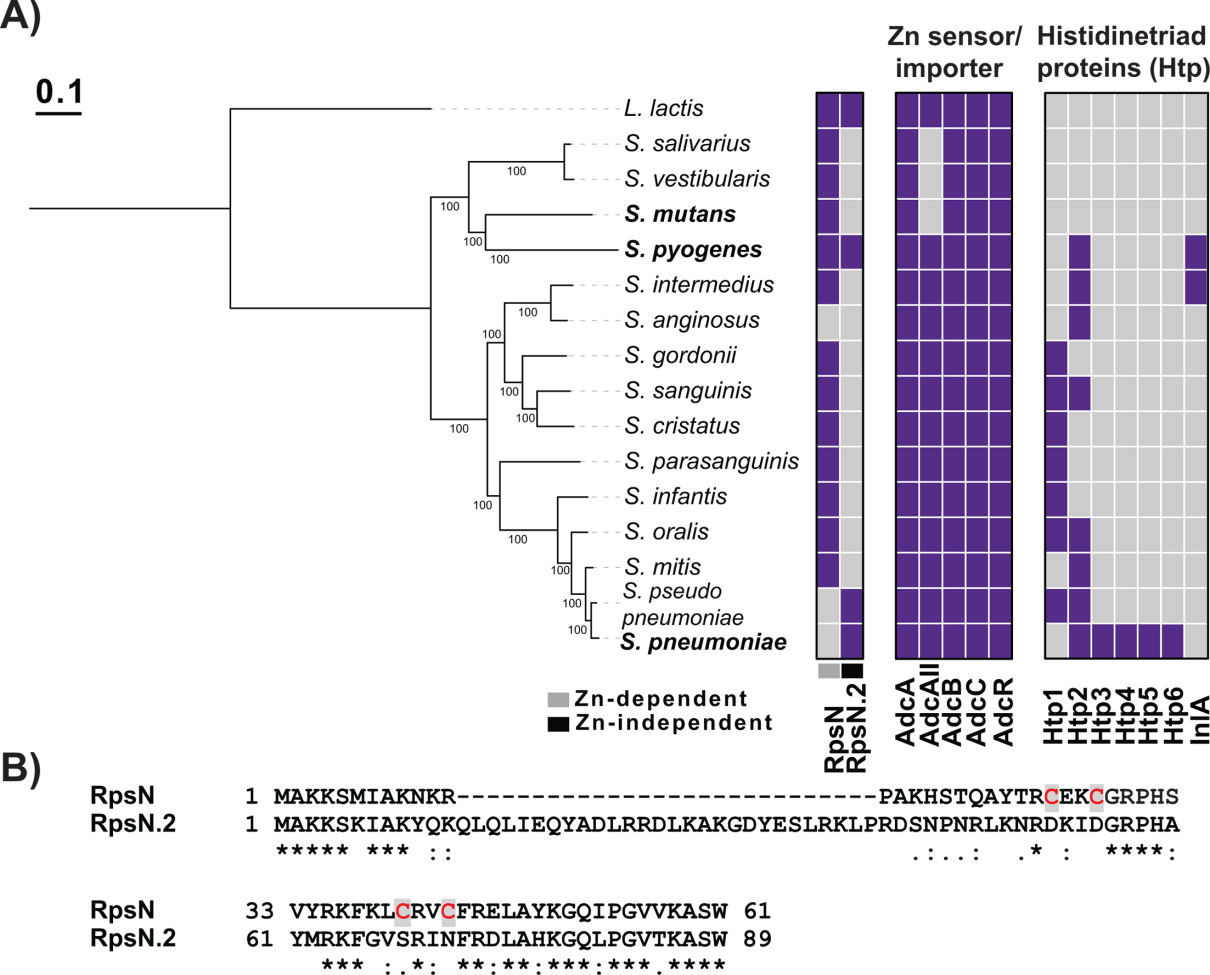
Comparative genomics of the AdcR regulon in commensal and pathogenic oral *Streptococcus* species. **(A)** The maximum likelihood core genome phylogeny of streptococcal reference genomes is shown on the left. *Lactococcus lactis* was used as an outgroup to root the tree. Bootstrap support values are shown below each branch, and the scale bar indicates the average number of amino acid substitutions per site. On the right, phyletic profiles of the AdcR regulon are displayed as presence/absence matrices: each row represents a *Streptococcus* genome, and each column corresponds to a single-copy orthologous group within the regulon. Genes present are shown in purple; absent genes are shown in gray. The first matrix shows the Zn-dependent RpsN and Zn-independent RpsN2 groups of the 30S ribosomal protein family S14. The second matrix corresponds to components of the AdcABCR system. The third matrix corresponds to ortholog group groups of proteins harboring the streptococcal histidine triad protein (Htp) domain (PFAM domain PF04270): the first six columns (Htp 1 to 6) correspond to groups of the same gene family, while the last column corresponds to the PF04270-domain containing leucine-rich internalin A-like gene family. (**B**) The amino acid sequence alignment of Zn-dependent RpsN and Zn-free RpsN.2 is shown. The Zn-binding cysteines in the CXXC2 motifs of RpsN are colored in red.

We found that the genes encoding the Zn-sensing transcription regulator *adcR* and the tripartite ABC-family Zn importer *adcABC* were present in the genomes of all the screened oropharyngeal commensal and pathogenic streptococci (Fig. 1A). These observations suggest that both commensal and pathogenic streptococci employ AdcR to monitor alterations in Zn availability and AdcABC to acquire extracellular Zn (Fig. 1A). However, the presence of the remaining components of the AdcR regulon varied between the commensal and pathogenic streptococci as well as among commensal streptococci. GAS, *S. pneumoniae*, and *S. pseudopneumoniae* possess all seven components of the AdcR regulon that include *adcR*, *adcA*, *adcB*, *adcC*, *adcAII*, *pht*, and *rpsN.2* (Fig. 1A). The cariogenic oral pathogen *S. mutans* and the commensal streptococci *S. salivarius* and *S. vestibularis* encode the least number of the AdcR regulon genes, as they contain only the genes encoding *adcR* and *adcABC* and lack *adcAII*, *pht*, and *rpsN.2* (Fig. 1A). All the other analyzed genomes of commensal streptococci had the gene encoding extracellular Zn binding *adcAII* and differing numbers of copies of genes encoding Pht proteins. GAS encodes two copies of *pht* genes, whereas pneumococci possess five different *pht* genes (Fig. 1A). The genomes of the commensal streptococci *S. intermedius, S. sanguinis, S. oralis*, and *S. pseudopneumoniae* have two copies of *pht* genes, whereas the genomes of *S. anginosus, S. gordonii, S. cristatus, S. parasanguinis, S. infantis*, and *S. mitis* encode a single copy of *pht*.

Intriguingly, with the exception of GAS, *S. pseudopneumoniae,* and *S. pneumoniae*, the *rpsN.2* gene encoding the alternative ribosomal S14 subunit was absent in the analyzed genomes of commensal streptococci and *S. mutans* (Fig. 1A; Fig. S1). GAS encodes two copies of the ribosomal S14 subunit, a Zn-containing *rpsN* and a Zn-free *rpsN.2*. The housekeeping S14 subunit RpsN contains a CXXC_2_ motif that binds Zn, and its activity is dependent on Zn occupancy (Fig. 1B). In contrast, RpsN.2 lacks CXXC_2_ motif, and the ribosomal binding by and activity of RpsN.2 are predicted to be Zn-independent (Fig. 1B; Fig. S1). Thus, it was proposed that RpsN.2 replaces Zn-containing RpsN in the ribosomes during Zn scarcity, which confers a survival advantage to GAS by sustaining protein synthesis and by releasing Zn bound to RpsN for other critical functions during Zn deficiency (28–30). Consistent with this, GAS upregulates the expression of *rpsN.2* during Zn scarcity, and *rpsN.2* is critical for CP resistance and GAS virulence (28, 30). Collectively, these observations indicate that pathogenic streptococci, such as GAS and *S. pneumoniae*, are equipped with the full repertoire of the AdcR regulon genes to evade Zn deficiency, and the gene encoding Zn-free ribosomal S14 subunit *rpsN.2* may confer fitness advantage to the pathogenic GAS to survive in Zn-limiting host niches.

### GAS upregulates *rpsN.2* expression during growth in human saliva

**Fig 2:**
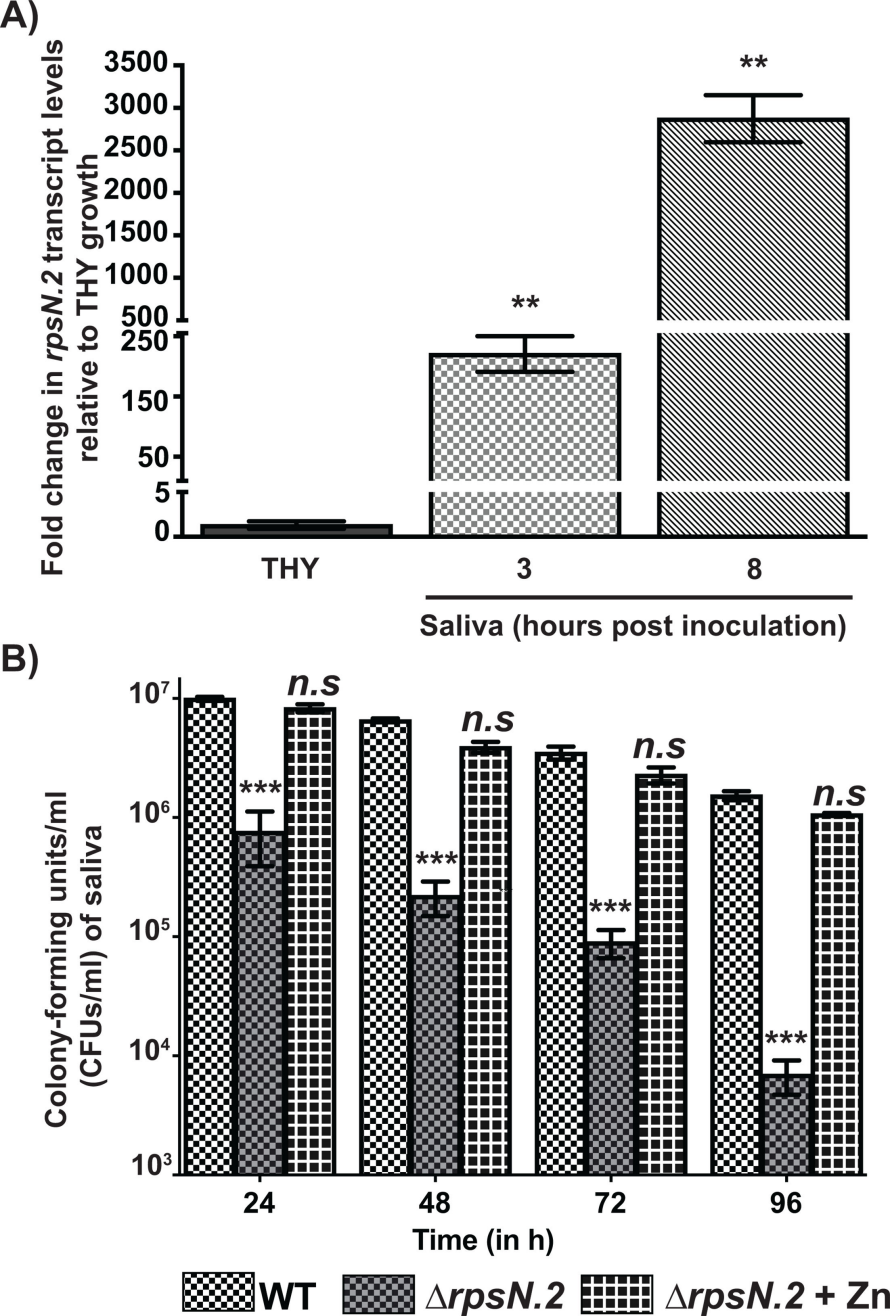
*rpsN.2* is critical for GAS survival in human saliva ex *vivo*. (**A**) WT GAS was grown in human saliva *ex vivo* and transcript levels of *rpsN.2* were assessed by qRT-PCR. GAS grown in THY was used as the reference bacterial growth. Fold change in *rpsN.2* transcript levels relative to reference is shown. Data graphed are mean ± standard deviation for three biological replicates grown on separate occasions. *P*-values (**, *P* < 0.001) were determined by comparison to THY growth and were derived from *t*-test. (**B**) The human saliva was inoculated with 10^3^ CFUs of either WT or ∆*rpsN.2* mutant. The ∆*rpsN.2* mutant was supplemented with 10 µM excess Zn (∆*rpsN.2* + Zn) to assess whether excess Zn aids *∆rpsN.2* mutant survival in saliva. Samples were collected at the indicated time points, serially diluted, plated, and GAS CFUs were enumerated. Three biological replicates grown on separate occasions were used, and the mean + standard deviation is shown. *P*-values (***, *P* < 0.0001, *n.s*—not significant) were determined by comparison to WT GAS control and were derived from Kruskal-Wallis test.

We previously demonstrated that the Zn concentration in healthy human saliva is relatively low (~540–580 nM) compared to the laboratory medium (~11 µM) (49, 50), and the levels are comparable to Zn scarcity *in vitro* (<1 µM). Given that GAS and other streptococcal species grow well in THY broth and GAS AdcR regulon genes are typically repressed during growth in THY, the Zn levels in THY represent non-toxic, Zn-replete growth condition. Since GAS persists in human saliva successfully, we hypothesized that GAS recruits *rpsN.2* during growth in Zn-sparse saliva *ex vivo*. To test this hypothesis, we compared the *rpsN.2* transcript levels in GAS grown in human saliva to GAS growth in Zn-replete THY. Consistent with our hypothesis, significant induction of *rpsN.2* expression was observed during growth in human saliva relative to THY, and the transcript levels of *rpsN.2* increased over time during GAS growth in saliva (Fig. 2A). These results indicate that GAS deploys *rpsN.2* during growth in Zn-limited human saliva, and *rpsN.2* may be critical for GAS survival in saliva. Conversely, the absence of the AdcR regulon genes, such as *rpsN.2*, is likely to make oropharyngeal commensal streptococci more sensitive to Zn deficiency.

### *rpsN.2* is critical for GAS survival in human saliva

Our previous studies showed that *rpsN.2* is critical for GAS virulence in mouse models of invasive infection (28). However, pharyngitis is the predominant form of GAS disease manifestations (51, 52), and GAS survival in human saliva is critical for disease transmission between individuals (53–55). Since the contribution of *rpsN.2* to GAS survival in human saliva is uncharacterized, we tested the hypothesis that *rpsN.2* is critical for GAS growth in Zn-scant saliva *ex vivo*. Consistent with our hypothesis, inactivation of the *rpsN.2* affected the survival of ∆*rpsN.2* mutant in saliva significantly compared with the WT GAS. The ∆*rpsN.2* mutant was defective in survival in saliva as early as 24 h post-inoculation (hpi), and the mutant strain was gradually cleared from saliva over 96 hpi (Fig. 2B), indicating that *rpsN.2* contributes significantly to GAS growth in Zn-sparse saliva. However, the defective survival of the *rpsN.2* mutant was restored to the WT GAS-like growth phenotype by supplementation with excess Zn (Fig. 2B), indicating that *rpsN.2* plays a key role in supporting GAS growth in Zn-limited host niches.

### *S. vestibularis* upregulates the expression of Zn importer during Zn scarcity

**Fig 3:**
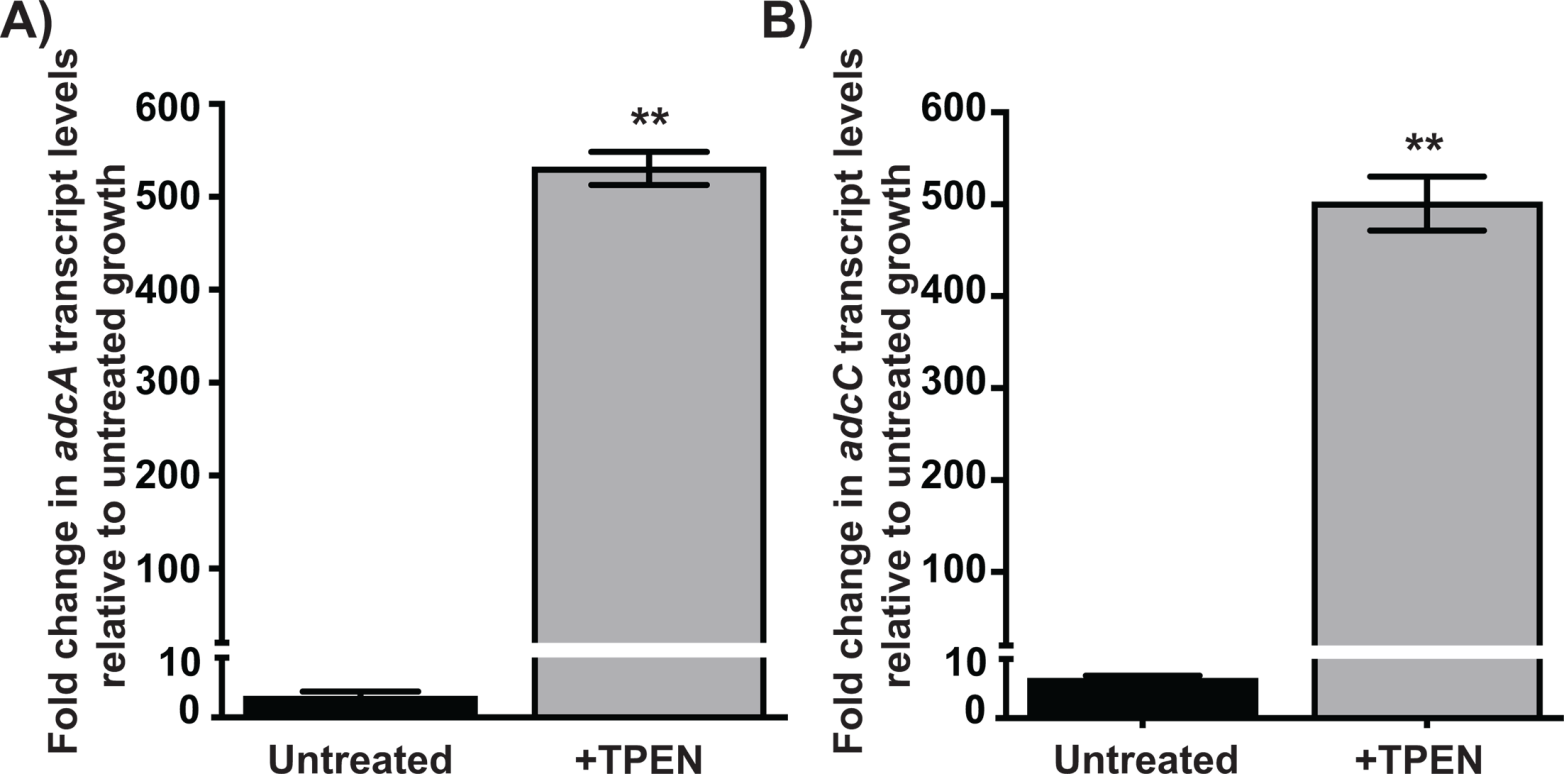
*S. vestibularis* deploys genes encoding putative Zn importer *adcA* **(A)** and *adcC* **(B)** during Zn scarcity. *S. vestibularis* was grown to late-exponential phase of growth (A_600_ ~ 1.0) in Zn-replete THY and incubated for 30 min with or without TPEN. Cells were collected, and transcript levels of *adcA* and *adcC* were measured by qRT-PCR. The TPEN untreated cells were used as a reference and fold change in transcript levels relative to reference is shown. Three biological replicates were grown on separate occasions, and the mean + standard deviation is shown. *P*-values (**, *P* < 0.01) were determined by comparison to reference and were derived from *t*-test.

To test whether commensal streptococci lacking *rpsN.2* and *pht* genes are more sensitive to Zn scarcity, we used *S. vestibularis,* which lacks the majority of the AdcR regulon genes (Fig. 1A), as a representative species of commensal *Streptococcus* and assessed its sensitivity to Zn limitation. First, we tested whether *S. vestibularis* upregulates the expression of the AdcR regulon genes in response to Zn scarcity similar to GAS (28, 30). We used streptococcal growth medium THY that contains ~11 µM Zn as the Zn-replete condition (50) and induced Zn scarcity by the addition of the Zn-specific chelator N,N,N′,N′-tetrakis-(2-pyridylmethyl) ethane-1,2-diamine (TPEN). Consistent with the Zn-specific regulation of the AdcR regulon in pathogenic streptococci, the expression of *adcA* and *adcC* was repressed in *S. vestibularis* grown in the absence of TPEN (Fig. 3A and B). However, the repression was relieved in the presence of TPEN, and robust upregulation of *adcA* and *adcC* was observed (>500-fold change) relative to cells grown in the absence of TPEN (Fig. 3A and B). These results indicate that *S. vestibularis* employs the AdcR regulon genes encoding the Zn importer *adcA* and *adcC* to negate Zn scarcity.

### *S. vestibularis* is more sensitive to Zn scarcity

**Fig 4:**
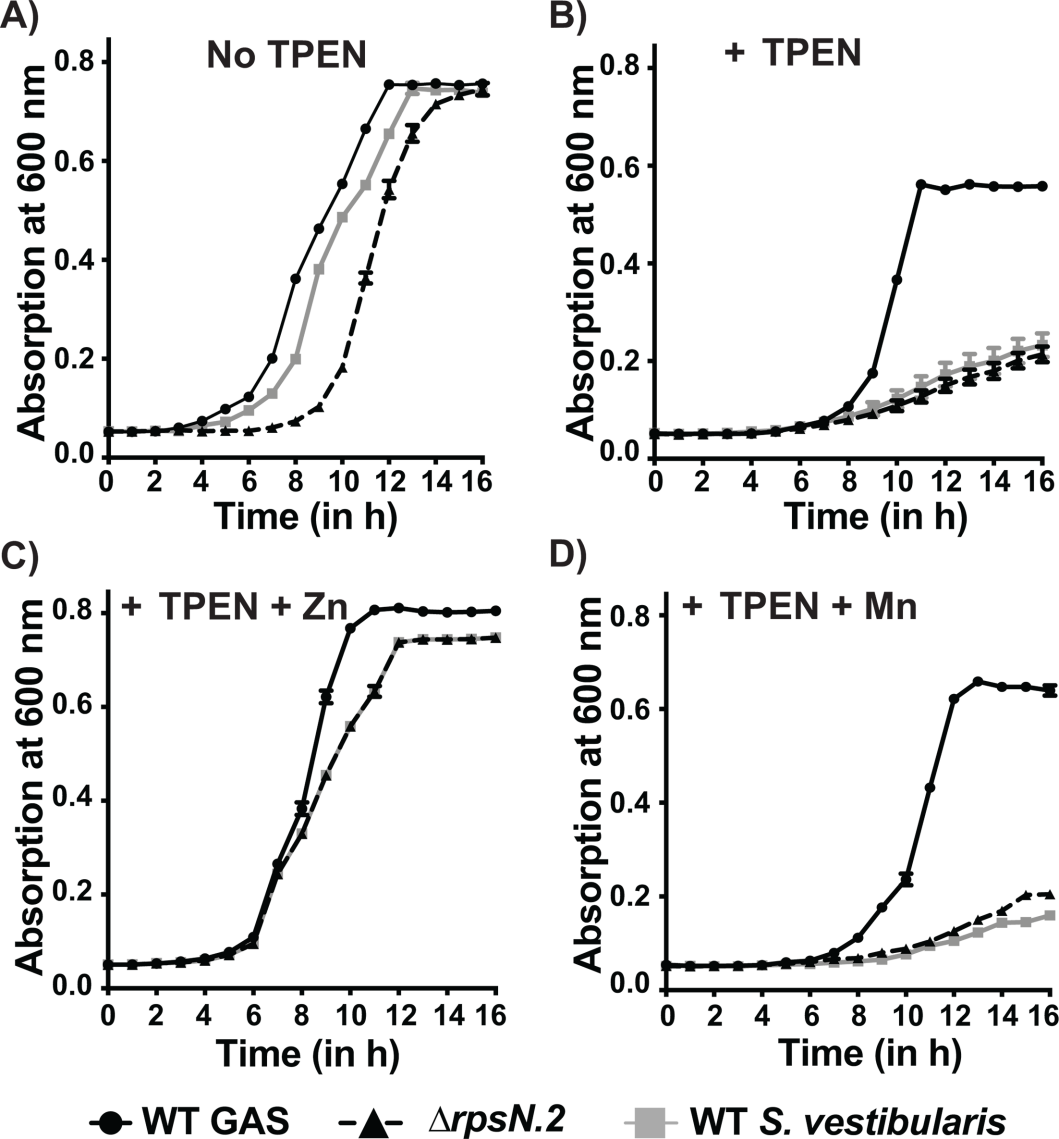
*S. vestibularis* is more sensitive to TPEN *in vitro* compared with GAS. The growth kinetics of WT GAS, ∆*rpsN.2* mutant GAS strain, and *S. vestibularis* grown in the presence or absence of 22.5 µM of TPEN is shown. Cells were grown in THY broth supplemented without (**A**) or with (**B**) TPEN. Bacterial growth was supplemented with either 10 µM Zn (**C**) or Mn (**D**) to test Zn-specific restoration of the growth phenotype. Growth was monitored by absorption at 600 nm in a microplate reader at indicated time points. Three biological replicates were grown on separate occasions, and the mean + standard deviation is shown.

Since *S. vestibularis* lacks components of the AdcR regulon (Fig. 1A), we hypothesized that *S. vestibularis* is more susceptible to Zn scarcity relative to GAS. To test this hypothesis, we compared the growth of commensal and pathogenic streptococci *in vitro* in the presence of the Zn chelator TPEN. The *rpsN.2*-inactivated GAS strain, the ∆*rpsN.2* mutant (28), was used to compare the growth kinetics of *S. vestibularis* and pathogenic *Streptococcus* lacking *rpsN.2*. The growth kinetics of all three strains were similar in the absence of TPEN (Fig. 4A). Although the growth kinetics of GAS was delayed in the presence of TPEN compared with that of unmodified THY, GAS sustained growth in the presence of TPEN (Fig. 4B). In contrast, both the *∆rpsN.2* mutant and *S. vestibularis* had increased sensitivity to TPEN relative to WT GAS and failed to grow in the presence of TPEN compared with GAS (Fig. 4B). However, the defective growth phenotype of the *∆rpsN.2* mutant and *S. vestibularis* in the presence of TPEN was restored to WT GAS-like growth by supplementation with excess Zn, whereas supplementation of non-Zn metal, such as Mn, failed to revert the defective growth phenotypes (Fig. 4C and D). Collectively, these results show that the oropharyngeal commensal *S. vestibularis* is more sensitive to Zn scarcity *in vitro* compared with GAS, and the lack of *rpsN.2* may contribute to the increased susceptibility of *S. vestibularis* to Zn deficiency.

### *S. vestibularis* is defective in survival in human saliva

**Fig 5:**
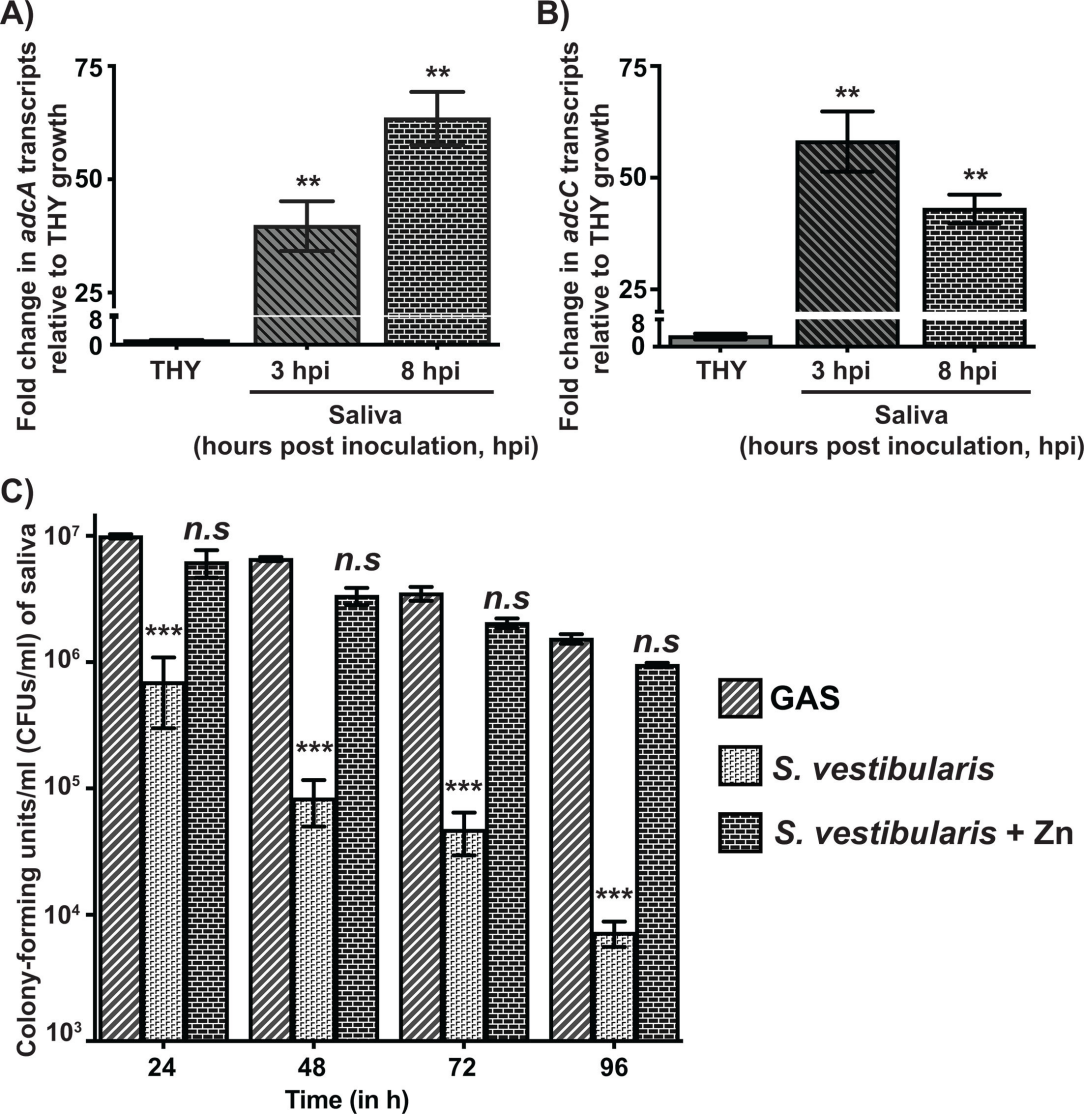
*S. vestibularis* lacking components of AdcR regulon is defective in survival in human saliva *ex vivo*. Transcript levels of *adcA* (**A**) and *adcC* (**B**) in *S. vestibularis* grown in human saliva *ex vivo* were assessed by qRT-PCR. Fold changes in transcript levels relative to *S. vestibularis* grown in Zn-replete THY are shown. Data graphed are mean ± standard deviation for three biological replicates grown on separate occasions. Statistical significance (**, *P* < 0.001) was determined by comparison to THY growth and was derived from *t*-test. (**C**) The human saliva was inoculated with 10^3^ CFUs of either WT GAS or *S. vestibularis. S. vestibularis* was supplemented with 10 µM excess Zn (*S. vestibularis* + Zn) to assess whether excess Zn aids growth in saliva. Samples were collected at the indicated time points, serially diluted, plated, and bacterial CFUs were enumerated. Three biological replicates grown on separate occasions were used, and the mean + standard deviation is shown. *P*-values (***, *P* < 0.0001, *n.s*—not significant) were determined by comparison to WT GAS and were derived from Kruskal-Wallis test.

*S. vestibularis* primarily colonizes the oral vestibular mucosal surfaces (46, 56) and is likely released into the saliva due to the shedding of the host epithelia and epithelia-associated bacteria. Consistent with this, *S. vestibularis* is present abundantly in human saliva and is a predominant member of the healthy human salivary microbiome (56, 57). Thus, it is likely that *S. vestibularis* survival in human saliva is critical for its colonization of the human oral cavity. However, the Zn requirement of *S. vestibularis* may differ from that of GAS, and salivary Zn levels may be sufficient to support *S. vestibularis* growth. Thus, we tested whether salivary Zn levels represent Zn-deficient growth conditions for *S. vestibularis* by measuring the transcript levels of *adcA* and *adcC* during growth in human saliva *ex vivo*. Compared with Zn-replete THY, *S. vestibularis* exhibited significant upregulation of *adcA* and *adcC* expression at 3 hpi and sustained higher expression levels of both genes at 8 hpi (Fig. 5A and B), a well-characterized streptococcal adaptive response to Zn scarcity (Fig. 3A) (15, 28–30). These observations indicate that similar to pathogenic GAS, *S. vestibularis* also encounters Zn-deficient growth conditions during survival in human saliva and employs *adcABC* to survive in Zn-sparse human saliva.

To determine whether salivary Zn levels influence the survival of *S. vestibularis* in saliva, we compared the growth of WT GAS and WT *S. vestibularis* in saliva. GAS reached a high population density (>10^7^ CFUs/mL) at 24 hpi and persisted at high levels (>10^6^ CFUs/mL) over 96 hpi in saliva *ex vivo* (Fig. 5C). In contrast, *S. vestibularis* showed a significant survival defect in saliva compared with WT GAS as early as 24 hpi, and gradual clearance of *S. vestibularis* occurred over 96 hpi (Fig. 5C). Intriguingly, the survival phenotype of *S. vestibularis* in saliva was similar to that of GAS ∆*rpsN.2* mutant (Fig. 2B and 5C), suggesting that the lack of *rpsN.2* contributes to the defective survival of *S. vestibularis* in saliva. However, the defective phenotype of *S. vestibularis* in saliva was restored to WT GAS levels by supplementation with Zn (Fig. 5C), indicating that Zn scarcity in saliva negatively impacts *S. vestibularis* survival in human saliva.

### *S. vestibularis* is more sensitive to CP-mediated Zn limitation

**Fig 6:**
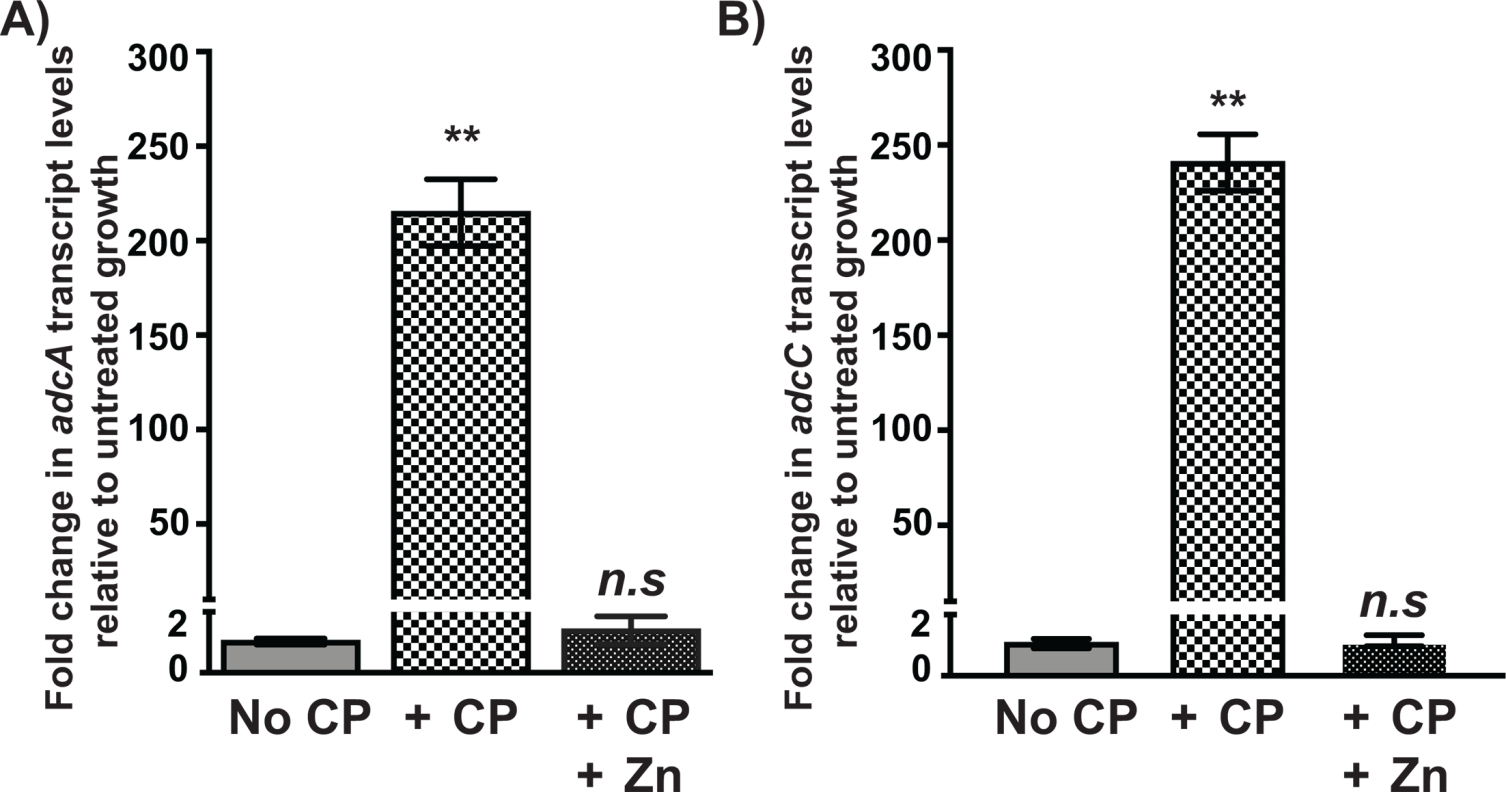
*S. vestibularis* upregulates the expression of genes encoding Zn importer in the presence of CP. *S. vestibularis* was grown to late-exponential growth phase (A_600_ ~1.0) in the presence or absence of WT CP. Transcript levels of *adcA* (**A**) and *adcC* (**B**) were assessed by qRT-PCR. Using the transcript levels in the unsupplemented growth (No CP) as reference, the fold change in transcript levels of *adcA* and *adcC* was determined and shown. Data graphed are mean ± standard deviation for three biological replicates grown on separate occasions. Statistical significance (**, *P* < 0.001, *n.s*—not significant) was determined by comparison to reference and was derived from *t*-test.

During pharyngitis, it is likely that CP is present in the saliva within the inflamed oral cavity, and CP-mediated Zn limitation may negatively impact the survival of oral commensal streptococci, such as *S. vestibularis*. To test this hypothesis, we assessed whether *S. vestibularis* senses Zn limitation in the presence of CP by measuring the transcript levels of *adcA* and *adcC* by qRT-PCR. Cells were grown to the late-exponential phase of growth (A_600_ ~1.0), incubated with or without CP for 30 min, and the expression of *adcA* and *adcC* was measured by qT-PCR. Consistent with our hypothesis, the expression of *adcA* and *adcC* was significantly upregulated in the presence of CP compared with unsupplemented growth (Fig. 6A and B). However, when cells were supplemented with excess Zn, *adcA* and *adcC* transcript levels reverted to basal levels in the presence of CP (Fig. 6), indicating that *S. vestibularis* senses CP-mediated Zn limitation and upregulates the expression of *adcA* and *adcC* to survive in the presence of CP.

**Fig 7:**
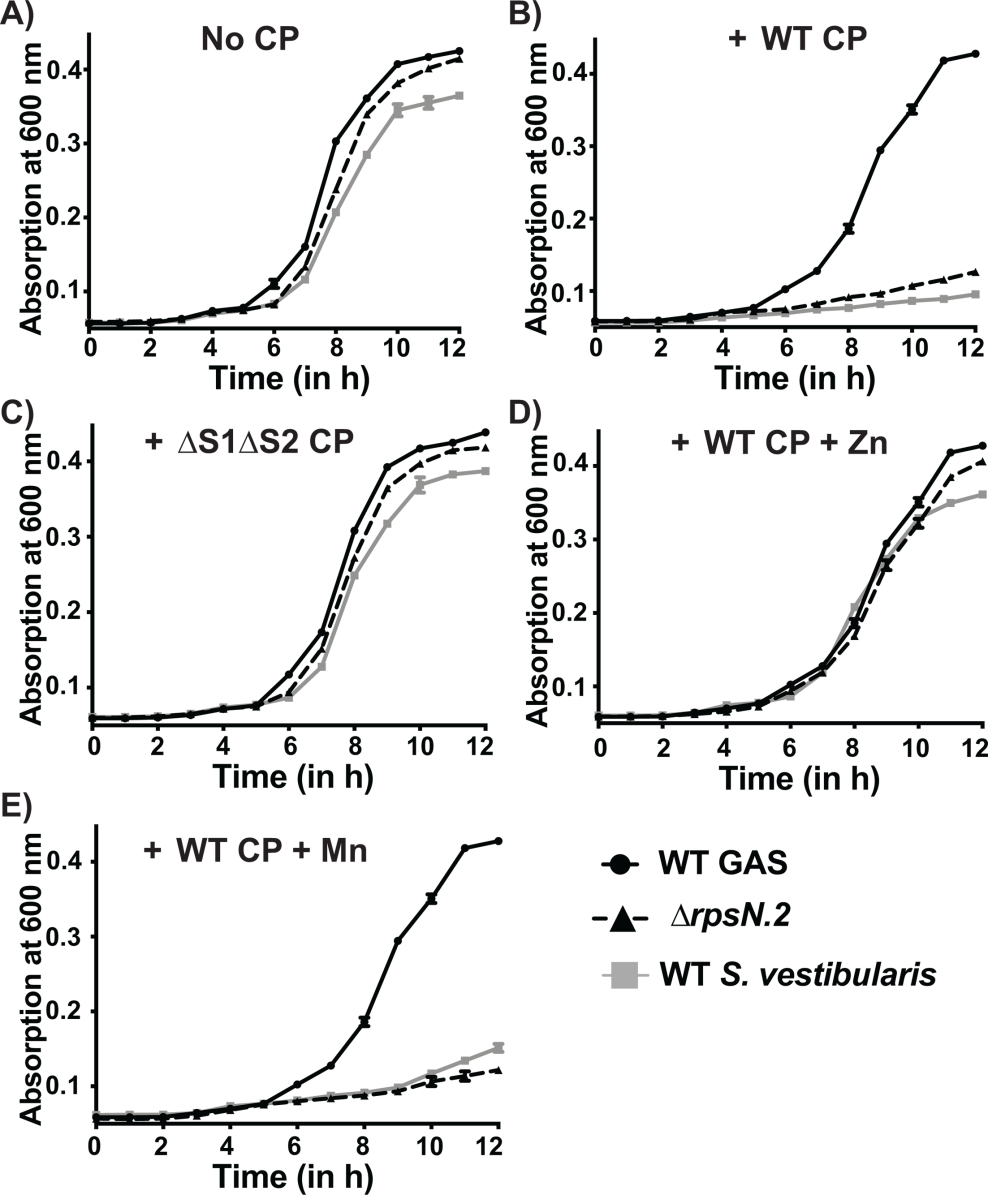
*S. vestibularis* is more sensitive to CP-mediated Zn limitation compared with GAS. The growth kinetics of WT GAS, ∆*rpsN.2* mutant GAS strain, and *S. vestibularis* grown in the presence or absence of 250 µg/mL of CP are shown. Cells were inoculated in THY-CP medium (38% (v/v) THY medium and 62% (v/v) CP buffer containing 20 mM Tris-HCl pH 7.5, 0.1 M NaCl, 10 mM β-mercaptoethanol, 3 mM CaCl_2_) supplemented without (**A**) or with (**B**) recombinant wild type (WT) CP. The ∆S1/∆S2 mutant (∆His_3_Asp site/∆His_6_ site) CP was used to assess whether the growth inhibition was due to metal limitation (**C**). Bacterial growth was supplemented with either 10 µM Zn (**D**) or Mn (**E**) to test metal-specific restoration of growth phenotype in the presence of CP. Growth was monitored by absorption at 600 nm in a microplate reader at indicated time points. Three biological replicates were grown on separate occasions, and the mean + standard deviation is shown.

We next hypothesized that the lack of components of the AdcR regulon renders *S. vestibularis* more sensitive to CP-mediated Zn limitation compared with GAS. To test this hypothesis, we compared the sensitivity of GAS and *S. vestibularis* to CP (250 µg/mL) by assessing the growth phenotype. The growth of GAS and *S. vestibularis* was similar in the absence of CP (Fig. 7A). However, *S. vestibularis* growth was inhibited by CP, whereas GAS growth remained unaffected in the presence of CP (Fig. 7B). Furthermore, the metal-binding defective ∆S1∆S2 CP mutant failed to inhibit *S. vestibularis* growth (Fig. 7C), indicating that *S. vestibularis* growth inhibition by CP is due to metal limitation. Consistent with this, the defective growth phenotype of *S. vestibularis* in the presence of CP was restored specifically by Zn supplementation (Fig. 7D), whereas excess Mn failed to confer CP resistance (Fig. 7E). Interestingly, the growth phenotype of *∆rpsN.2* mutant of GAS exhibited CP sensitivity similar to that of *S. vestibularis* and the growth kinetics of ∆*rpsN.2* in the presence of CP was comparable to that of *S. vestibularis* (Fig. 7), suggesting that *rpsN.2* is a key component of GAS defense against CP-mediated Zn limitation. Collectively, these findings demonstrate that *S. vestibularis* is more sensitive to CP-mediated Zn limitation compared with GAS.

## DISCUSSION

Our comparative analyses of the AdcR regulon between oropharyngeal pathogenic and commensal streptococci showed that oropharyngeal pathogens, such as GAS, are genetically endowed with a multitude of molecular arsenals and mechanisms to survive in Z- limiting host niches. The adaptive mechanisms include the deployment of ABC-family Zn importer AdcABC, extracellular Zn binding membrane protein AdcAII, cell wall-associated polyhistidine triad proteins (Phts/Htps), and intracellular Zn-sparing alternative ribosomal S14 subunit, RpsN.2. Contrary to this, with the exceptionofr Zn importer AdcABC, the oropharyngeal commensal streptococci lack several components of the AdcR regulon. Importantly, the Zn-independent ribosomal S14 subunit, *rpsN.2,* is absent in all the analyzed genomes of oropharyngeal commensal streptococci, suggesting its critical contribution to streptococcal pathogenesis (Fig. 1; Fig. S1). Consistent with this, our results show that GAS is equipped to survive in Zn-limiting host environments as it withstands Zn scarcity during growth *in vitro*, survives in Z-scarcet human saliva *ex vivo*, and evades CP-mediated Zn limitation. Conversely, the commensal streptococcus, *S. vestibularis*, is more sensitive to Zn deficiency, defective in survival in saliva, and unable to overcome CP-mediated Zn sequestration. Collectively, our findings indicate that *rpsN.2* plays a major role in GAS evasion of host-imposed Zn scarcity and likely confers survival advantage to the pathogen over commensal streptococci in Zn sparse host environments or in inflamed tissues.

Zn sequestration by CP in inflamed tissues and its significance to infection control are well documented (9–15, 20, 28, 31, 58). However, several studies showed that pathogenic bacteria overcome CP-imposed Zn scarcity and survive successfully in Zn-limited host environments (9–15, 20, 28, 31, 58). Conversely, the members of healthy microbiota are shown to be more susceptible to Zn deficiency, which leads to alterations in microbiome composition and associated negative consequences on host health. The Zn acquisition by high-affinity Zn importers of pathogenic bacteria is often thought to confer the ability to outcompete CP for Zn and support pathogen survival in Z- sparse host niches (11, 14, 15, 20, 31, 49). However, our analyses indicate that oropharyngeal commensal streptococcal genomes also encode high-affinity Zn importers, which share as high degree of sequence and structural similarity with that of pathogenic bacteria. Consistent with this, *S. vestibularis* significantly upregulates the expression of Zn importer *adcABC* in response to Zn scarcity (Fig. 3, 5 and 6), indicating an adaptive response similar to pathogenic bacteria (29). Thus, it is unlikely that Zn importer alone confers survival advantage to the pathogen over commensal during Zn scarcity. Alternatively, our findings suggest that *rpsN.2* may represent the first line of GAS defense to evade Zn scarcity and provide fitness advantage to pathogenic GAS to survive in Zn-limiting host environments. In accordance with this, *Bacillus subtilis* employs alternative Zn-free ribosomal subunits as the first response to Zn scarcity even preceding the upregulation of the expression of Zn importers (59, 60). Similarly, our previous findings showed that upregulation of *rpsN.2* and *adcABC* constitutes the primary GAS adaptive responses to Zn deficiency, whereas *adcAII, phtD*, and *phtY* play a relatively lesser role in GAS survival in Zn-limited host niches, CP resistance, and GAS virulence (28).

The Zn-free paralogs of ribosomal subunits have been implicated in the bacterial adaptive responses to Zn scarcity (61). *Streptomyces coelicolor* encodes seven pairs of Zn-containing and Zn-free paralogs of ribosomal subunits and upregulates the expression of Zn-free paralogs during Zn scarcity (62). Similarly, *B. subtilis* has two copies of the genes encoding L31, L33, and S14 ribosomal subunits, a Zn-dependent and a Zn-free forms (59, 60, 63–65). Furthermore, *Mycobacterium tuberculosis* genome contains five alternative ribosomal proteins that have a Zn-containing primary ribosomal subunits (66–68). The alternative ribosomal proteins are upregulated during Zn scarcity and critical for bacterial survival in Zn-limiting growth conditions. The replacement of Zn-containing paralogs with Zn-free paralogs in ribosomes confers survival advantage to the bacteria during Zn scarcity by mobilizing the intracellular Zn associated with Zn-containing paralogs and by sustaining protein synthesis via the remodeled ribosomes containing Zn-free ribosomal subunits (59, 60). However, GAS specifically recruits the Zn-free paralog of ribosomal S14 subunit, *rpsN.2*, during Zn scarcity (28, 30), which underscores the significance of the alternative S14 subunit to GAS pathogenesis.

Although our findings underscore the significance of RpsN.2 in the survival of pathogenic streptococci during Zn scarcity, they also raise questions about the contribution of Zn-dependent RpsN to bacterial survival. In the absence of experimental evidence comparing the efficiency of RpsN and RpsN.2 in ribosome assembly, translation kinetics, and disassembly from the ribosome, it is difficult to delineate their roles in bacterial survival in the host. One possibility is that pathogenic streptococci encounter varying Zn availability ranging from Zn scarcity to Zn toxicity during different stages of infection (69). Thus, the presence of Zn-dependent RpsN is likely to facilitate bacterial survival during Zn sufficiency or surplus, whereas the deployment of Zn-free RpsN.2 and the associated release of Zn from RpsN may promote the survival of pathogenic streptococci during Zn scarcity. Intriguingly, the characterization of alternative Zn-free ribosomal subunits in *M. tuberculosis* indicated an additional important role for RpsN.2 in pathogenic bacterial adaptation to host-induced Zn deficiency. The binding of Zn-free ribosomal subunits to ribosomes during Zn scarcity leads to the recruitment of bacterial translation hibernation factors to the ribosome (67, 70). The binding of hibernation factors to the ribosomes resulted in slower translation kinetics during mild Zn scarcity to stalled inactive ribosomes during severe Zn deficiency (67, 70). These observations suggest that Zn-dependent ribosomal factors, such as RpsN, promote the assembly of ribosomes with superior translation efficiency than their Zn-free counterparts. Additional investigations are required to determine whether streptococcal RpsN.2 is involved in promoting bacterial survival by slowing down or stalling ribosomes during Zn scarcity.

Our analyses also revealed differences in the adaptation of pathogenic streptococci to Zn limitation. Although *S. pneumoniae* also encodes the alternative S14 subunit *rpsN.2*, the expression of *rpsN.2* remains unaltered during Zn scarcity, indicating that *rpsN.2* may not contribute to pneumococcal evasion of host-imposed Zn limitation (33). Intriguingly, the AdcR regulon of the cariogenic pathogen *S. mutans* resembles that of oral commensal streptococci (32) and only contains the core components of the AdcR regulon (Fig. 1A). However, the successful survival of *S. mutans* in saliva and colonization in the human oral cavity are suggestive of distinct adaptive strategies and/or lesser Zn dependence of *S. mutans* for survival compared with that of GAS or pneumococci (71).

In summary, our analyses revealed the unique adaptive strategies employed by oropharyngeal streptococcal pathogen GAS to evade host-imposed alterations in Zn availability and survive in the host. Specifically, except for *S. mutans*, the presence of alternative ribosomal S14 subunit *rpsN.2* appears to be unique in oropharyngeal pathogenic streptococcal genomes. Thus, we propose that *rpsN.2* represents a pathogen-specific mechanism to overcome host-imposed Zn limitatio,n and *rpsN.2* may confer survival advantage to the pathogen over oropharyngeal commensal streptococci by facilitating their survival during infection-induced reduction in Zn availability.

## MATERIALS AND METHODS

### Bacterial strains, plasmids, and growth conditions

The bacterial strains and plasmids used in this study are listed in Table S5. Strain MGAS10870 is a representative of serotype M3 strains that cause invasive infections (72). The whole genome of MGAS10870 has been fully sequenced and has WT sequences for all major regulatory genes (72). To compare the adaptive responses of pathogenic and commensal streptococcal species to Zn scarcity, *S. vestibularis* ATCC49124 strain at hand was used as a representative commensal streptococcus species. *Escherichia coli* DH5α was used as the host for plasmid cloning, whereas *E. coli* BL21(DE3) was used for recombinant protein overexpression. GAS strains were routinely grown in Todd-Hewitt broth containing 0.2% (w/v) yeast extract (THY; Difco) or Trypticase soy agar containing 5% sheep blood (bovine serum albumin [BSA]; Becton, Dickinson). For growth studies in the presence of CP, THY-CP medium was prepared by adding 38% (vol/vol) THY to 62% (vol/vol) CP medium (20 mM Tris HCl [pH 7.5], 100 mM NaCl, 10 mM β-mercaptoethanol, and 3 mM CaCl_2_). *E. coli* strains DH5α and BL21(DE3) were grown in lysogeny broth (LB; Teknova). Overnight cultures of GAS were inoculated in fresh medium with an initial absorption of 0.03 at A_600_. Bacterial growth was monitored by measuring the optical density at A_600_ with a microplate reader. Chloramphenicol and ampicillin were added to the cultures to a final concentration of 5 or 80  µg/mL, respectively, when required.

### Comparative genomics of Zn scarcity-related genes

The reference, complete assembly of the 17 species was downloaded from the NCBI using the following url: https://www.ncbi.nlm.nih.gov/datasets/genome/?taxon=1301&reference_only=true. The annotated proteomes from these assemblies were compared in an All-vs-All run of MMSeqs2 version 16-747c6 at maximal sensitivity (option -s 7.5) (73). Only pairwise alignments with at least 25% sequence identity over a minimum of 60% of the length of the shorter protein were retained. Gene families were defined as clusters within the resulting sequence similarity network, identified by the Markov Clustering Algorithm (MCL) version 14-137 with an inflation parameter of 2 (74). In the network, edge weights were calculated using the bitscore ratio of pairwise alignments: (bitscore _A vs B_ + bitscore _B vs A_) / (bitscore _A vs A_ + bitscore _B vs B_). Best bidirectional hits between each pair of genomes were identified from the all-vs-all comparison, using the lowest E-value as the selection criterion. Orthologous groups were defined as connected components in the network of best-bi directional hits. The 700 core single-copy orthologous groups (with a unique member in each of the 17 genomes) were further aligned with mafft version 7.525 with the option --maxiterate 1,000 --localpair for high accuracy (75). A maximum-likelihood core genome phylogeny was derived from the concatenated alignment of the 700 single-copy core proteins using IQ-TREE multicore version 2.2.2.6, employing 1,000 ultrafast bootstraps and the LG + F + I + R4 model of amino-acid substitution, which was assessed as the best fit according to the Bayesian Information Criterion (76). The phylogeny was further rooted using *Lactococcus lactis* as the outgroup of the *Streptococcus* species and visualized on the Interactive Tree of Life Browser (77). Histidine triad proteins were identified by searching the proteomes with the PFAM HMM model PF04270 (streptococcal histidine triad protein) using hmmsearch from the HMMER suite (version 3.3.2), applying the—cut_ga threshold option (78). Zn-dependent S14 ribosomal proteins were identified by scanning ribosomal protein sequences for the presence of the CXXC motif using regular expression matching. Zn-independent ribosomal proteins were defined as those belonging to the same gene families as Zn-dependent ribosomal proteins but lacking the CXXC motif. Components (AdcA, AdcAII, AdcB, AdcC, AdcR) of the Adc system were already described in *S. pyogenes*. We then generated a presence/absence phyletic matrix (species vs orthogroup) using orthogroups from protein families containing the Zn scarcity-related proteins. The phyletic matrix was visualized using the ComplexHeatmap R package version 2.15.4 (79).

### Transcript level analyses

GAS strains were grown under the indicated growth conditions and incubated with two volumes of RNAprotect (Qiagen) for 10  min at room temperature. Cells were harvested by centrifugation, and the cell pellets were snap-frozen in liquid nitrogen. RNA isolation and purification were performed using RNeasy kit (Qiagen) according to the manufacturer’s protocol. The concentration of RNA was measured using a NanoDrop 8000 instrument (Thermo Fisher Scientific). cDNA was synthesized from 2 µg of total RNA using Superscript III (Invitrogen), and quantitative PCR (qPCR) was performed using SYBR Green Q-PCR master mix (GenDEPOT) with an AB1 7500 fast system (Applied Biosystems). The specificity of the reaction was verified with melt curve analysis. Comparison of transcript levels was performed using the threshold cycle (Δ*C_T_*) method of analysis using *tufA* as the endogenous control gene (30). The primers used for reverse transcriptase quantitative PCR (qRT-PCR) are listed in Table S6.

### Collection of human saliva

Saliva from adult volunteers was collected on ice under a protocol approved by the Institutional Review Board at Houston Methodist Research Institute (approval number Pro00003833) using the method described previously with minor modifications (30). The saliva was clarified by centrifugation at 23,000 × *g* for 1 h, followed by filtration through a 0.22-μm-pore-size membrane filter (Corning, NY). Pooled saliva was stored frozen at −20°C. Saliva from at least four donors was pooled to minimize the potential effects of donor variation.

### Bacterial survival studies in saliva

The ability of GAS strains to grow and persist in human saliva was evaluated as described previously (80, 81). Briefly, human saliva was collected from healthy volunteers and pooled as described above. GAS was grown overnight in Todd-Hewitt broth supplemented with 0.2% yeast extract (THY; BD Biosciences, Sparks, MD), diluted 1:100 with fresh THY, and grown to the growth phase indicated above. The bacterial cells were pelleted, washed twice with sterile PBS, and suspended in saliva at ∼1 × 10^3^ CFU/mL. Aliquots were removed at the time points indicated in the figures. Samples were serially diluted 10-fold in sterile PBS and plated in duplicate on Trypticase soy agar plates supplemented with 5% sheep blood (BD Biosciences). The plates were incubated overnight, and colonies were counted to determine the number of CFU. All incubations were at 37°C with 5% CO_2_. Each experiment was performed in triplicate on three separate occasions.

### Recombinant CP purification

Overexpression and purification of recombinant human WT or mutant calprotectin (CP) were carried out, as previously described (15). Overnight cultures of BL21(DE3) containing the coding sequences of *S100a8* or *S100a9* in plasmid pET15b were diluted 1:50 in fresh LB medium and grown at 37°C until the A_600_ reached 0.4 to 0.6. Protein overexpression was induced by the addition of 1 mM isopropyl β-d-1-thiogalactopyranoside (IPTG), and cells were grown at 37°C for an additional 4 h. Equal amounts of cell pellets (by weight) containing *S100a8* and *S100a9* overexpression plasmids were suspended in buffer A (20  mM Tris HCl [pH 8.0], 0.1 M NaCl, 10 mM β-mercaptoethanol, 1 mM EDTA, and 0.5% Triton X-100) supplemented with a protease inhibitor pellet (Roche). Cells were lysed with a cell lyser (Microfluidics), and inclusion bodies containing S100A8 and S100A9 proteins were fractionated by centrifugation at 21,000  ×  *g* for 30  min. The pellet was resolubilized by suspending it in buffer containing 50 mM Tris HCl (pH 8.0), 100 mM NaCl, 10 mM β-mercaptoethanol, and 4 M guanidine hydrochloride. Refolding of the proteins was achieved by overnight dialysis using a 10 kDa cutoff membrane against the base buffer containing 20  mM HEPES (pH 8.0). After three rounds of dialysis, the final dialyzed sample was centrifuged at 21,000  ×  *g* for 30  min and filtered with a 0.22 µm syringe filter. The S100A8/S100A9 heterodimer was separated by ion-exchange chromatography using a Mono-Q column (GE Lifesciences) preequilibrated with base buffer (20  mM HEPES [pH 8.0], 10  mM β-mercaptoethanol) and eluted using a salt gradient of 0 to 300  mM NaCl. The sample was further purified by size exclusion chromatography using Superdex 26/600 200 kDa (GE Lifesciences). Finally, the metal-free form of the S100A8/S100A9 heterodimer was prepared with a two-step dialysis as follows: first, against storage buffer containing 20 mM HEPES (pH 8.0), 100 mM NaCl, 10 mM β-mercaptoethanol, and 10 mM EDTA and, second, against chelexed storage buffer without EDTA. The S100A8/S100A9 heterodimer was concentrated using a YM-10 filter to a final concentration of 10 mg/mL, and flash frozen aliquots were stored at –80°C until used.

### GAS growth studies with CP

A GAS inoculum of 10^5^ CFUs/mL was inoculated into THY-M broth supplemented with CP buffer. The recombinant purified CP was added to the starter culture. After 6 h incubation, cells were collected, serially diluted, plated, and subjected to bacterial survival analyses by enumerating CFUs. Samples were analyzed in triplicate, and at least two different CP preparations were used.

## SUPPLEMENTAL MATERIAL

Supplemental material iai.00442-25-s0001.pdf Fig. S1 and Tables S5 and S6.

Table S1 iai.00442-25-s0002.xlsx Reference genomes used in this study.

Table S2 iai.00442-25-s0003.xlsx Orthologous groups of the AdcR regulon gene products.

Table S3 iai.00442-25-s0004.xlsx Prevalence of RpsN and RpsN2 copies within the *Streptococcus* genus and species.

Table S4 iai.00442-25-s0005.xlsx All-vs-all BLAST results for rpsN and rpsN.2 sequences.

ASM does not own the copyrights to Supplemental Material that may be linked to, or accessed through, an article. The authors have granted ASM a non-exclusive, world-wide license to publish the Supplemental Material files. Please contact the corresponding author directly for reuse.
