# Supplementary material for: A zinc-free alternative ribosomal protein RpsN.2 confers survival advantage to group A streptococcus during Zn scarcity
Source: Infect Immun. 2025 Nov 18;93(12):e00442-25. doi: 10.1128/iai.00442-25 (PMC12707111; doi:10.1128/iai.00442-25)
Supplement: Supplemental material — Fig. S1 and Tables S5 and S6. [file iai.00442-25-s0001.pdf]

Supplementary Information

Supplementary figure S1

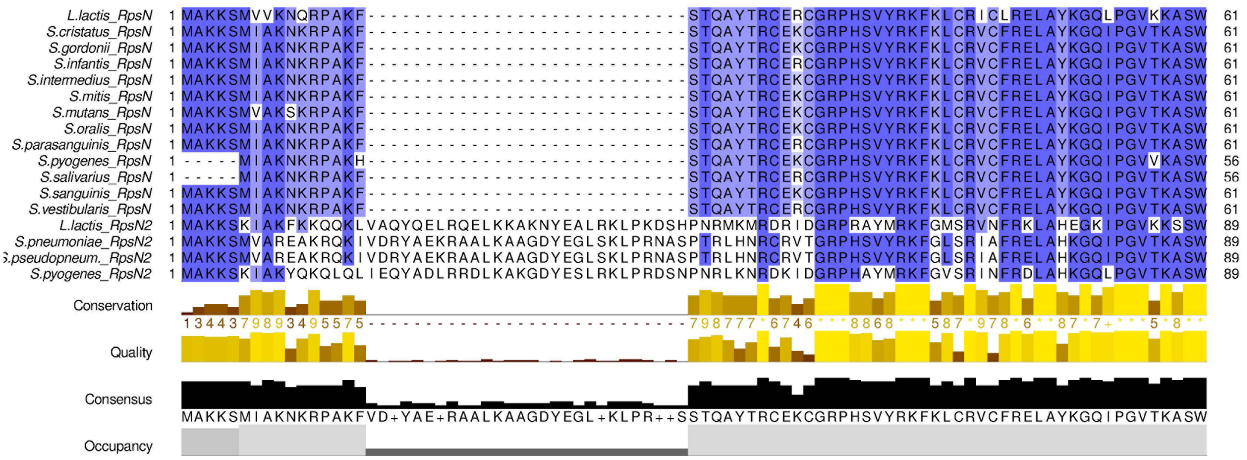

Supplementary figure S1. Amino acid sequence alignment of RpsN and RpsN.2 in oral pathogenic and commensal streptococci. The amino acids highlighted in blue showing the conserved amino acids among the RpsN and RpsN.2.

Supplementary table S5

**Strains**	**Description**	**Reference**
WT GAS	Invasive isolate MGAS10870, serotype M3	(1)
WT *Streptococcus vestibularis*	Invasive isolation, ATCC49124	(2)
∆*rpsN.2*	Isogenic mutant strain that has the inframe deletion of rpsN.2 in parental serotype MGAS10870	(3)
E. coli BL21 (DE3)	Host strain for protein overexpression, calprotectin (CP)	(3)
**Plasmids**		
pET15b	Overexpression vector used to produce tag-free S100A8 and S100A9, Amp resistant	(3)

Supplementary table S6. Primers used in this study

**Primer**	**Sequence 5' – 3'**	**Purpose**	**Reference**
rpsN.2 qRTFwd	ACGCTGACCTTCGTCGTG	5' primer for *rpsN.2* qRTPCR	(3)
rpsN.2 qRTRev	GTTTGGATTTGAATCACG	3' primer for *rpsN.2* qRTPCR	(3)
tufA GAS qRTFwd	CAACTCGTCACTATGCGCACAT	5' primer for tufA qRTPCR	(3)
tufA GAS qRTRev	GAGCGGCACCAGTGATCAT	3' primer for *tufA* qRTPCR	(3)
tufA S. vestibularis qRTFwd	CCAGAACGTGACACTGACAA	5' primer for *tufA* qRTPCR	This study
tufA S. vestibularis qRTRev	AACAACACCACGGTCGATAC	3' primer for *tufA* qRTPCR	This study
adcA S. vestibularis qRTFwd	CCTGAAACAGGAAGCGATG ATA	5' primer for *adcA* qRTPCR	This study
adcA S. vestibularis qRTRev	CACTCACCAGACCAATCAGAAA	3' primer for *adcA* qRTPCR	This study

adcC S. vestibularis qRTFwd	TTGTGAAGTCTGGCCGTTATC	5' primer for *adcC* qRTPCR	This study
adcC S. vestibularis qRTRev	TCCCACATACCTACAGACTCAA	3' primer for *adcC* qRTPCR	This study

1. Andreini C, Banci L, Bertini I, Rosato A. 2006. Zinc through the three domains of life. J Proteome Res 5:3173–3178. doi: 10.1021/pr0603699.

2. Whiley RA, Hardie JM. Streptococcus vestibularis sp. nov. from the human oral cavity. Int. J. Syst. Bacteriol. 38: 335-339, 1988.

3. Makthal, Nishanth, et al. "Group A Streptococcus AdcR regulon participates in bacterial defense against host-mediated zinc sequestration and contributes to virulence." Infection and Immunity 88.8 (2020): 10-1128.
